# The Economic Burden of Heart Conditions in Brazil

**DOI:** 10.5935/abc.20180104

**Published:** 2018-07

**Authors:** Bryce Stevens, Lynne Pezzullo, Lara Verdian, Josh Tomlinson, Alice George, Fernando Bacal

**Affiliations:** 1 Deloitte Access Economics Pty Ltd, Austrália; 2 Instituto do Coração (InCor) - HC-Faculdade de Medicina da USP, São Paulo, SP – Brazil

**Keywords:** Cardiovascular Diseases/economics, Hypertension, Heart Failure, Myocardial Infarction, Atrial Fibrillation

## Abstract

**Background:**

Heart conditions impose physical, social, financial and health-related quality of life limitations on individuals in Brazil.

**Objectives:**

This study assessed the economic burden of four main heart conditions in Brazil: hypertension, heart failure, myocardial infarction, and atrial fibrillation. In addition, the cost-effectiveness of telemedicine and structured telephone support for the management of heart failure was assessed.

**Methods:**

A standard cost of illness framework was used to assess the costs associated with the four conditions in 2015. The analysis assessed the prevalence of the four conditions and, in the case of myocardial infarction, also its incidence. It further assessed the conditions’ associated expenditures on healthcare treatment, productivity losses from reduced employment, costs of providing formal and informal care, and lost wellbeing. The analysis was informed by a targeted literature review, data scan and modelling. All inputs and methods were validated by consulting 15 clinicians and other stakeholders in Brazil. The cost-effectiveness analysis was based on a meta-analysis and economic evaluation of post-discharge programs in patients with heart failure, assessed from the perspective of the Brazilian Unified Healthcare System (Sistema Unico de Saude).

**Results:**

Myocardial infarction imposes the greatest financial cost (22.4 billion reais/6.9 billion USD), followed by heart failure (22.1 billion reais/6.8 billion USD), hypertension (8 billion reais/2.5 billion USD) and, finally, atrial fibrillation (3.9 billion reais/1.2 billion USD). Telemedicine and structured telephone support are cost-effective interventions for achieving improvements in the management of heart failure.

**Conclusions:**

Heart conditions impose substantial loss of wellbeing and financial costs in Brazil and should be a public health priority.

## Introduction

Heart conditions impose physical, social, financial and health-related quality of life limitations on individuals. These conditions result in an economic burden and impact on society due to expenditures on healthcare treatment, productivity losses from employment impacts, costs of providing formal and informal care, and lost wellbeing. Circulatory diseases presently represent the biggest health burden worldwide, accounting for over 17 million deaths every year; this represents half of all noncommunicable disease deaths.^1^

At the 2016 World Congress of Cardiology & Cardiovascular Health, the Mexico Declaration for Circulatory Health was signed by leading global organisations committed to improving circulatory health and reducing deaths and disability from heart diseases and stroke around the world. This is aligned with a clear target, set by the World Health Organization (WHO) and signed by country signatories, of reducing deaths from noncommunicable disease by 25 per cent by 2025.^1^ Our analysis identifies the current burden heart conditions have on Brazil and consequently the potential economic benefits that could result from addressing it.

This study aims to assess the economic (health system and productivity) impact of four heart conditions in Brazil, providing estimates of the annual cost for the year 2015: hypertension (HTN), myocardial infarction (MI), atrial fibrillation (AF) and heart failure (HF). This study also analyzes the cost-effectiveness of two interventions for HF: telemedicine (TM) and structured telephone support (STS).

## Method

This research is part of a larger study of the Latin American region, with country-specific results also identified for Mexico, Chile, Peru, Venezuela, Colombia, Ecuador, Panama and El Salvador. These results for Brazil were presented at ISPOR Vienna (November 2016) and the World Cardiovascular Congress (June 2016).

### Cost of illness

The analysis was based on estimating the prevalence, incidence, loss of wellbeing, health system and productivity losses attributed to the four heart conditions. Total cost estimates were adjusted based on the comorbidity between conditions. Underpinning the study was a literature search that used search terms associated with the country, region, epidemiology and economic impact of the four heart conditions. Sources included PubMed, government, healthcare and patient organization websites, and general internet search engines.

### Prevalence/incidence of conditions

The sources used for estimating the prevalence or incidence are outlined in Table 1. Whenever possible, Brazil specific rates were used. All estimates were checked with stakeholders interviewed for the project. Identified rates were applied to projections from the United Nations World Population Prospects.^2^

**Table 1:** Number of people with the four heart conditions in Brazil, 2015

Condition	Number of people	Percentage of the adult population*
HF	2 845 722	2.0
MI	334 978	0.2
AF	1 202 151	0.8
HTN	44 526 201	31.2
Total conditions	48 909 052	34.3
Total persons with any condition (i.e. accounting for comorbidities)	45 658 048	32.0

* Percentage reflects the evidence from studies among populations aged 20 years and over. HF: heart failure; MI: myocardial infarction; AF: atrial fibrillation; HTN: hypertension.

### Loss of wellbeing

Disability weights were based on the WHO Global Burden of Disease studies^3,4^ as shown in Table 2. These were then multiplied by the prevalence estimates to identify the years lost to disability for 2015. Years lost to life were based on reported mortality for each condition.

**Table 2:** Financial cost of heart conditions in Brazil, 2015 (millions of reais)

Category	HF	MI	AF	HTN	Total (unadjusted)	Total (adjusted for comorbidities)^^^
Health system costs	14 469	16 119	3 697	1 098	35 382	35 382
	65%	72%	94%	14%	63%	63%
Productivity losses	7 663	6 257	225	6 927	21 071	20 858
	35%	28%	6%	86%	37%	37%
Income forgone by individuals*	3 528	4 540	156	2 063	10 287	10 196
	16%	20%	4%	26%	18%	18%
Income forgone by businesses*	333	403	31	4 378	5 145	5 050
	2%	2%	1%	55%	9%	9%
Opportunity cost of informal care by family/friends	2 404	196			2 600	2 596
	11%	1%			5%	5%
Tax revenue forgone by government**	1 399	1 117	37	486	3 039	3 016
	6%	5%	1%	6%	5%	5%
Total cost	22 132	22 375	3 921	8 025	56 454	56 241

Results in millions of reais.

* The result from absenteeism, reduced employment participation, and premature mortality.

** Due to reduced income of individuals with heart conditions and their carers.

^ Comorbidity totals do not sum to the total of the individual conditions as one person can have more than one condition and the interaction between conditions causes the total estimate of the four conditions together to vary. HF: heart failure; MI: myocardial infarction; AF: atrial fibrillation; HTN: hypertension.

### Health system costs

The discharges and average length of stay for each of the conditions^5^ were combined with cost estimates for each of the four condition categories^5^ to estimate each condition’s burden on the health system as a share of all conditions treated. This was then combined with an estimate of total relevant health expenditure for Brazil^6^ to result in the cost of treating each of the four conditions. Health costs were estimated from the perspective of health care payers, i.e. both public and private payers. Cost breakdowns were based on those reported for Brazil.^7^ This method allows us to reflect most appropriately the impacts based on the number, length of stay and cost intensity of each condition for Brazil specifically. However, data on condition-specific health expenditures are not available for other components of the health system (e.g. primary care). Accordingly, each condition’s share of total health system expenditure was assumed to be the same as its share of total hospital expenditure.

### Productivity losses

Consistent with the ‘full or near-full employment’ criterion,^a^ a human capital approach to the estimation of productivity losses was adopted. Calculations involving productivity losses were based on employment rates by age-gender groups. It was assumed that those with heart conditions were, in the absence of the condition, as likely to be employed as others in their corresponding age-gender group. Forgone wage income was based on wage data for Brazil.^7^

Absenteeism was associated with all of the conditions. For HF it was estimated as 12.66 days for those with NYHA III/IV and 3.04 days per year for those with NYHA I/II.^8^ Absenteeism was estimated as 3.03 days per year^8^ for HTN, 75 days per year for those admitted to hospital^9^ with MI, and 2.1 days per year^10^ for AF. Reduced employment participation, where individuals are no longer able to be employed due to their condition, was identified for both HF and MI, but not for AF or HTN. For HF, there was 13% lower employment participation rate (based on those with coronary heart disease).^11^ The study also showed increased withdrawal of unemployed people from the labor force, especially those aged below 60 years and those engaged in manual work. For MI, there was a 21% lower employment participation [based on those with acute coronary syndrome (ACS) five years after an event].^12^ As the lower employment participation rates in both the coronary heart disease and ACS studies were based on populations in developed countries, these rates were adjusted by the observed rates of reduced employment participation for those with disability in Europe and Latin America, as reported by the Organization for Economic Cooperation and Development (OECD).^13^

Forgone income due to premature death was based on mortality statistics for each condition and the otherwise expected life expectancy according to WHO life tables.^14^ The anticipated number of years of life left to live by the deceased individual was multiplied first by employment rates and then by the average weekly wage for men and women respectively. The productivity discount rate for future earnings was 5.25% based on the difference between wage growth and inflation (using the annualized average for both over the past five years). The present value of future wages was based on the five-year average real growth rate.^15^

Informal care costs were identified for both HF and MI. For HF, each individual was provided an estimated 6.7 hours of informal care per week.^16^ While there are a variety of sources for this parameter, the study chosen was the most robust methodologically and provided a similar estimate to what could be derived from a study in Latin America.^17^ For MI, based on a study of coronary heart disease patients, informal care hours were estimated to be 279 hours per year per patient.^18^

Taxation revenue foregone was based on the average income tax rate for a single individual and the average indirect tax rate according to the OECD.^19,20^ The estimated income tax liability was applied to the estimated total value of forgone earnings to determine the value of taxation lost. An adjustment was also applied for the number working in the ‘informal economy’ which is likely to reduce the taxation revenue collected. Exchange rates between USD and the local currency were based on the average of the daily exchange rates from the International Monetary Fund from January 2015 to November 2015.

### Comorbidities

**Figure 1:**
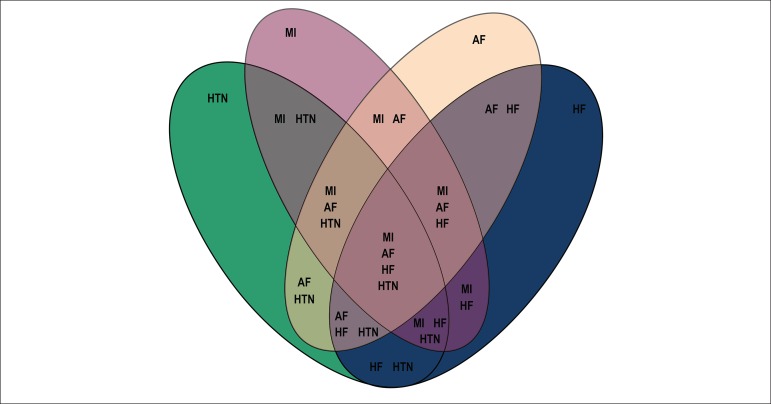
Potential comorbidity combinations accounted for. HF: heart failure; MI: myocardial infarction; AF: atrial fibrillation; HTN: hypertension.

As multiple conditions could affect one person simultaneously, the total cost of the four conditions was estimated by reviewing literature^21-23^ that identified the number of individuals with two, three or four concomitant conditions as outlined in Figure 1. Where literature did not outline the concomitant rates between each of the four conditions, the sources were extrapolated until all combinations were derived.

### Cost-effectiveness analysis for HF

To undertake the analysis, a targeted literature review was carried out to identify either published cost-effectiveness studies which could be adapted to the Brazilian context, or literature which could inform the design of, and inputs to, a cost-effectiveness model. The review identified a relatively recent network meta-analysis and cost-effectiveness analysis of TM and STS programs after discharge in patients with HF, conducted by the National Institute for Health Research in 2013.^24^ This study was therefore used as the basis for a cost-effectiveness analysis of STS and TM from the perspective of the Sistema Unico de Saude.

### Model structure

**Figure 2:**
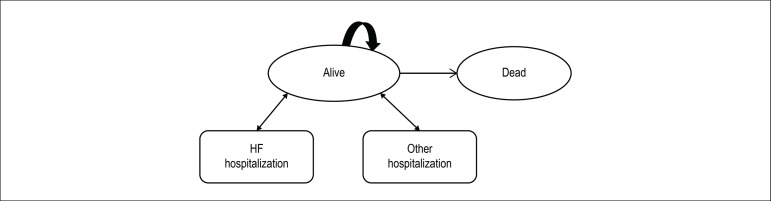
Markov model for recently discharged heart failure (HF) patients.

A Markov model was constructed in TreeAge Pro©2015 to evaluate the cost-effectiveness of STS and TM compared with standard care (SC) for a hypothetical cohort of patients discharged in the last 28 days following HF-related hospitalizations. The model as shown in Figure 2 considered two different permanent health states, ‘alive at home’ and ‘dead’ as well as two temporary health states for ‘hospitalized due to HF’ and ‘hospitalized for all other causes’. The model is based on monthly cycles with half-cycle corrections.

### Time horizon, duration and discount rate

As HF is a life-long condition after onset, the model captured a lifetime horizon of 30 years with patients progressing through the model until they either died or reached the end of the 30-year time horizon. It was assumed that the interventions of STS, TM and SC were provided for the full duration of the time horizon, outside of hospitalization. Both health system costs and quality-adjusted life-years (QALYs) were discounted at an annual rate of 5%.

### Data sources

#### Efficacy estimates

The monthly probability of death with SC following a non-fatal hospitalization was based on data from the CHARM study,^25^ which followed 7,572 patients for a period of 38 months and showed the mortality risk to be the highest after hospital discharge, then decreasing over time. The mean number of HF-related and other (all cause) hospitalizations were based on a published meta-analysis^26^ and estimated by the National Institute for Health Research.^24^

Effectiveness parameters relating to risks of death and hospitalization for STS and TM interventions were based on the hazard ratios for all-cause mortality, all-cause hospitalizations and HF-related hospitalizations during the treatment period. The hazard ratios were estimated from the network meta-analysis by the National Institute for Health Research.^24^

#### Health state utilities

Health state utilities for SC, STS and TM treatment approaches were based on the previous economic model of TM strategies conducted in a published meta-analysis,^26^ which used utilities of 0.612 and 0.662 for SC and STS/TM groups, respectively. As with previous economic analyses, a negative adjustment of 0.1 was applied to account for the disutility associated with HF-related hospitalizations.^24^

#### Resource utilization and costs

STS and TM consist of three main units of healthcare resources:

- devices and equipment within the patient’s home, which include the device hub, peripherals and communication costs;
- maintenance/monitoring in the STS or TM center; and
- medical care units to deal with events or alerts, such as GP or nurse visits, or hospital-based outpatient visits.

The units of resources making up the components of SC, STS and TM were based on the published literature, and unit costs were obtained from DATASUS, the Brazilian Ministry of Health’s data department.

## Results

### Cost of illness for heart failure, myocardial infarction, atrial fibrillation and hypertension

The four heart conditions were estimated to affect approximately 45.7 million people in Brazil, 32.0% of the adult^b^ population. After adjusting for comorbidities, heart conditions were conservatively estimated to result in a financial cost of 56.2 billion reais (17.3 billion USD) in 2015 in Brazil. Of this, approximately 62.9% was health system cost. In 2015, the burden of these four conditions comprised approximately 5.5% of total national healthcare expenditure.

### Prevalence/incidence

HTN has the highest prevalence of the four conditions, followed by HF. As outlined in Table 1 there were 48.9 million conditions affecting 45.7 million people (some people have more than one condition).

### Economic impact

MI imposes the greatest financial cost, followed by HF, HTN and, finally, AF. Table 2 outlines the cost per condition by bearer of cost, demonstrating that each condition impacts individuals, government and society differently. Health costs make up the majority of expenditure for HF, MI and AF, reflecting the nature of Brazil’s health system.

Table 3 shows that HTN has the lowest cost per case and MI the highest. While the costs per case seem quite small for HTN, they reflect the total cost of the condition divided by the total number of people with the condition; whether they are receiving treatment or not. This per person cost should be considered in this ‘average’ context, rather than reflecting the actual health costs incurred for someone receiving treatment.

**Table 3:** Financial cost of heart conditions in Brazil per case, 2015 (reais)

	HF	MI	AF	HTN
Health system cost per case	5 085 (65%)	48 118 (72%)	3 075 (94%)	25 (14%)
Productivity cost per case	2 693 (35%)	18 678 (28%)	187 (6%)	156 (86%)
Total financial cost per case	7 777	66 797	3 262	180

HF: heart failure; MI: myocardial infarction; AF: atrial fibrillation; HTN: hypertension.

### Loss of wellbeing

In addition, the heart conditions included impose a substantial wellbeing loss as outlined in Table 4. Of the 3.2 million disability adjusted life years (DALYs), adjusted for comorbidities, there are 1.9 million healthy years lost due to disability (YLD) and over 1.3 million years of life lost due to premature mortality (YLL).

**Table 4:** Loss of wellbeing of heart conditions in Brazil, 2015

Condition	YLDs	YLLs	DALYs
HF	270 806 (14%)	251 136 (18%)	521 941 (16%)
MI	2 128 (0.1%)	1 112 469 (80%)	1 114 597 (34%)
AF	269 014 (14%)	28 237 (2%)	297 251 (9%)
HTN	1 380 312 (72%)		1 380 312 (42%)
Total (unadjusted)	1 922 260	1 391 842	3 314 102
Total (adjusted for comorbidities)^	1 901 386	1 340 453	3 241 838

HF: heart failure, MI: myocardial infarction, AF: atrial fibrillation, HTN: hypertension.

^ Comorbidity totals do not sum to the total of the individual conditions as one person can have more than one condition and the interaction between conditions causes the total estimate of the four conditions together to vary. YLDs: years lost due to disability; YLLs: years of life lost due to premature mortality; DALYs: disability-adjusted life-years.

### Cost-effectiveness analysis for heart failure

#### Base case result

Over the 30-year time horizon, the estimated discounted cumulative costs for the TM and STS interventions were 50,098 and 44,038 reais higher than SC, respectively, but generated an additional 1.91 and 1.63 QALY, respectively. This resulted in an estimated incremental cost-effectiveness ratio (ICER) of 26,437–81,984 reais/QALY and 27,281 reais/QALY for TM and STS, respectively, compared to SC, noting a willingness to pay (WTP) threshold of 27,328 reais/QALY. The threshold was based on one to three times the GDP per capita of Brazil.^6c^ The incremental net monetary benefit was 1,688 reais for TM vs SC and 77 reais for STS vs SC (Table 5).

**Table 5:** Base case result

	SC	TM	STS
Total costs (reais)	5 832	55 930	49 870
Total QALYs	3.99	5.89	5.61
Net monetary benefit	103 306	104 994	103 382
Incremental costs (reais)		50 098	44 038
Incremental QALYs		1.89	1.61
Incremental cost (reais) per QALY		26 437	27 281
Incremental net monetary benefit		1 688	77

QALY: quality-adjusted life-year; SC: standard care; TM: telemedicine; STS: structured telephone support.

#### Multivariate sensitivity analysis

An alternative multivariate scenario analysis was carried out where the costs of TM and STS were varied as well as the health state utilities. In this scenario, the costs of the interventions were increased by 20% and the health state utilities for health states for the strategies were assumed to be the same as those for SC. The results of this scenario analysis are presented in Table 6, which shows that the ICER increases from 26,437 to 41,123 reais/QALY for TM vs SC, and increases from 27,281 to 40,309 reais/QALY for STC vs SC.

**Table 6:** Multivariate sensitivity analysis

	SC	TM	STS
Total costs (reais)	5,832	68 891	58 538
Total QALYs	3.99	5.45	5.30
Incremental costs (reais)		60 059	52 706
Incremental QALYs		1.46	1.31
Incremental cost (reais) per QALY		41 123	40 309

QALY: quality-adjusted life-year; SC: standard care; TM: telemedicine; STS: structured telephone support.

Assuming a WTP threshold of 27,328- 81,984 reais/QALY as above, the cost-effectiveness analysis suggests that TM and STS may be cost-effective treatment options for the management of patients with HF.

## Discussion

Our analysis provides the inaugural estimate on the cost of the four conditions across Brazil. By analysing four conditions concurrently in a common framework, we were able to identify the total impact and the impacts of the conditions relative to each other. We have identified that, while MI has significant acute care costs, it does not have as significant informal care costs as HF or HTN. Conversely, HF, while not having as significant acute care costs as MI, has significant productivity losses. While HTN has a low health cost per person, it has a significant total cost due to the large number of people with the condition. Our analysis demonstrates that these conditions can have a large productivity and wellbeing impact beyond their health system costs, which is an important finding from a societal perspective. If policymakers focus only on health costs of a condition, or the relative cost of care per person, they may miss the broader impact that these conditions have across the economy, and the true cost once other fiscal impacts are taken into account.

While the study has focused on using administrative datasets for health costs, as they are more likely to be reflective of cost allocation by payers, the datasets themselves may not reflect real costs for each condition. For example, the coding and reporting of conditions is subject to clinicians’ individual judgement in nominating the underlying cause, active condition, or chronic condition as the primary condition, and this choice can change the reporting of attributable impacts. A systematic review and meta-analysis of administrative databases for HF identified that datasets do not capture a quarter of cases,^27^ while a systematic review of electronic medical data for AF identified that there was a disproportionate focus on inpatient data and additional research incorporating outpatient codes, and electrocardiogram data are required to correctly identify the presentations of AF.^28^ Therefore, while the costs reported are reflective of current clinical judgement and administrative reporting, the cost allocation attributable to each condition can continue to be improved.

In attributing the relative severity of conditions, their treatment and the impact on related conditions should be considered. Treatment of one of these conditions could alleviate the future development of another costed condition, and the detailed relationships between conditions are still being established. For example, while HTN is understood to be a common risk factor for heart conditions, there is a growing body of evidence that suggests AF is associated with MI.^29^ Therefore, addressing AF could alleviate future cases of MI and the corresponding cost attributed to MI.

The primary limitation in this study was comprehensive data availability. There are three key assumptions in the methodology that had to be made and could impact the results, which the reader should keep in mind. First, our health cost estimates are driven by reported hospital statistics for each of the conditions. This is likely to be more appropriate for conditions that have significant acute care management (e.g. MI), but it may under-represent the true cost of conditions that have a greater emphasis on primary care or pharmaceutical management, such as HTN. Second, common to all productivity estimates using a human capital approach, the unemployment rate for Brazil may or may not be sufficiently low to incur a permanent productivity loss. A loss in productivity due to heart conditions from a societal perspective will only equate to a loss in productivity to the economy under the condition that the economy is at the non-accelerating inflation rate of unemployment, so any reduction in hours worked due to illness cannot be replaced in the longer term by employing or increasing hours of other substitute workers. Thirdly, although TM and STS were found to provide beneficial effects in reducing all-cause mortality for recently discharged HF patients, in the original study,^24^ these results were statistically inconclusive. While this uncertainty around estimates was assessed in the sensitivity analysis, these strategies will need to be re-examined as new evidence emerges.

## Conclusion

This study has found that heart conditions impose significant financial and wellbeing impacts across Brazil, with the four conditions costing $56.2 billion reais in 2015 alone. Prevention or better management of heart conditions could result in significant benefits both in improved wellbeing and economic savings. Telemedicine and structured telephone support are cost effective mechanisms for achieving improvements in the management of heart failure.

The study was supported by funding from the Novartis Group. The authors are solely responsible for its content.

## References

[1] World Heart Federation (2016). The Brazil Declaration.

[2] Department of Economic and Social Affairs. World Population Prospects; 2015.

[3] Salomon JA, Haagsma JA, Davis A, de Noordhout CM, Polinder S, Havelaar AH (2015). Disability weights for the Global Burden of Disease 2013
study. Lancet Glob Health.

[4] World Health Organization.(WHO) (2004). Global burden of disease 2004 update.

[5] Brasil (2015). Ministério da Saúde. Informações de
Saúde. DATASUS Tecnologia da Informação a Serviço do
SUS.

[6] The World Bank (2015). World Development Indicators.

[7] World Health Organization. (WHO) (2015). Global Health Expenditures Database.

[8] Vuong TD, Wei F, Beverly CJ (2015). Absenteeism due to functional limitations caused by seven common
chronic diseases in US Workers. J Occup Environ Med.

[9] Dennis C, Houston-Miller N, Schwartz RG, Ahn DK, Kraemer HC, Gossard D (1988). Early return to work after uncomplicated myocardial infarction.
Results of a randomized trial. JAMA.

[10] Rohrbacker NJ, Kleinman NL, White SA, March JL, Reynolds MR (2010). The burden of atrial fibrillation and other cardiac arrhythmias
in an employed population: associated costs, absences, and objective
productivity loss. J Occup Environ Med.

[11] Kruse M, Sørensen J, Davidsen M, Gyrd-Hansen D (2009). Short and long-term labour market consequences of coronary heart
disease: a register-based follow-up study. Eur J Cardiovasc Prev Rehabil.

[12] Osler M, Mårtensson S, Prescott E, Carlsen K (2014). Impact of gender, co-morbidity and social factors on labour
market affiliation after first admission for acute coronary syndrome. A
Cohort Study of Danish Patients 2001-2009. PLoS One.

[13] OECD Sickness (2010). Disability and Work: Breaking the Barriers: A Synthesis of Findings
across OECD Countries, OECD Publishing, Paris.

[14] World Health Organisation (2013). (WHO) Life Tables.

[15] International Labour Organization (ILO) (2015). Yearly Indicators. ILOSTAT Database.

[16] Gure T, Kabeto M, Blaum C, Langa K (2008). Degree of disability and patterns of caregiving among older
americans with congestive heart failure. J Gen Intern Med.

[17] Araujo D, Tavares LR, Verissimo R, Ferraz MB, Mesquita ET (2005). Cost of heart failure in the unified health
system. Arq Bras Cardiol.

[18] Liu JLY, Maniadakis N, Gray A, Rayner M (2002). The economic burden of coronary heart disease in the
UK. Heart.

[19] OECD Personal Income Tax Statistics 2014.

[20] OECD Consumption Tax Trends 2014.

[21] Chow G V (2012). Epidemiology of arrhythmias and conduction disorder in older
adults. Clin Geriatr Med.

[22] Picariello C, Lazzeri C, Attanà P, Chiostri M, Gensini GF, Valente S (2011). The impact of hypertension on patients with acute coronary
syndromes. Int J Hypertens.

[23] DeFrances CJ, Lucas CA, Buie VC, Golosinskiy A (2008). Epidemiology and risk profile of heart failure. Natl Health Stat Report.

[24] Pandor A, Thokala P, Gomersall T, Baalbaki H, Stevens JW, Wang J (2013). Home telemonitoring or structured telephone support programmes
after recent discharge in patients with heart failure: systematic review and
economic evaluation. Health Technol Assess.

[25] Solomon SD, Dobson J, Pocok S, Skali H, McMuray JJV, Granger CB (2007). Influence of nonfatal hospitalization for heart failure on
subsequent mortality in patients with chronic heart failure. Circulation.

[26] Klersy C, Silvestri AD, Gabutti G, Raisaro A, Curti M, Regoli F (2011). Economic impact of remote patient monitoring:an integr ated
economic model deriv ed from ameta-analysis of randomized controlled trials
inheart failure. Eur J Heart Fail.

[27] McCormick N, Lacaille D, Bhole V, Anvina-Zubieta JA (2014). Validity of Heart Failure Diagnoses in Administrative Databases:
A Systematic Review and Meta-Analysis. PLoS One.

[28] Jensen PN, Johnson K, Floyd J, Heckbert SR, Carnahan R, Dublin S (2012). A systematic review of validated methods for identifying atrial
fibrillation using administrative data. Pharmacoepidemiology and Drug Safety.

[29] Violi F, Soliman EZ, Pignatelli P, Pastori D (2016). Atrial fibrillation and myocardial infarction: a systematic
review and appraisal of pathophysiologic mechanisms. J Am Heart Assoc.

